# Cutaneous Melanoma with Brain Metastasis: Report of 193 Patients with New Observations

**DOI:** 10.1371/journal.pone.0156115

**Published:** 2016-05-23

**Authors:** Alenka Gugger, Raymond L. Barnhill, Burkhardt Seifert, Silvia Dehler, Holger Moch, Claire Lugassy, Ewerton Marques-Maggio, Elisabeth J. Rushing, Daniela Mihic-Probst

**Affiliations:** 1 Institute of Surgical Pathology, University Hospital Zurich, Zurich, Switzerland; 2 Departments of Pathology and Translational Research, Institut Curie, Paris, France; 3 Department of Biostatistics, Epidemiology, Biostatistics and Prevention Institute, University of Zurich, Zurich, Switzerland; 4 Cancer Registry Zurich and Zug, Institute of Surgical Pathology, University Hospital Zurich and Epidemiology, Biostatistics and Prevention Institute, University of Zurich, Zurich, Switzerland; 5 Institute of Neuropathology, University Hospital Zurich, Zurich, Switzerland; University of Connecticut Health Center, UNITED STATES

## Abstract

**Background:**

Brain metastasis is a common endpoint in patients suffering from malignant melanoma. However, little is known about factors that predispose to brain metastases.

**Objective:**

We performed a retrospective clinical and pathological investigation of melanoma patients with brain metastases in order to better characterise this patient population.

**Methods:**

193 melanoma patients with brain metastasis histologically diagnosed between 1990 and 2015 at the University Hospital Zurich were retrospectively identified and further specified for sex, age at diagnosis and detection of brain metastasis, and localisation. In addition, data were extracted regarding the subtype of primary melanoma, Breslow tumour thickness, Clark Level, mutation status, extent of metastatic spread and history of a second melanoma.

**Results:**

We found a significant male predominance (n = 126/193; 65%; p < 0.001). Breslow tumour thickness showed a wide range from 0.2 to 12.0 mm (n = 99; median 2.3 mm). 14 of 101 melanomas (14%) were classified as T1, thereof 11 (79%) were found in men. In 32 of 193 patients (17%), the primary melanoma was unknown.

**Conclusions:**

Of special interest in our series is the high incidence of male predominance (79%) in cases of thin metastasing melanoma (14%), implicating genetic or epigenetic (hormonal) gender differences underlying tumour progression. Additionally, the high percentage of unknown primary melanoma (17%), at least partly representing completely regressed melanomas, indicates the importance of immune surveillance in melanoma progression.

## Introduction

The development of brain metastases, reported to occur in up to 25% of patients with cancer, is a common and devastating complication of many systemic malignancies [1]. Cutaneous melanoma is a highly aggressive cancer with a propensity for brain metastases. The reported incidence of cerebral metastases is variable and dependent on whether the source is clinical or autopsy data, the patient population, and other factors. A study analysing 700 melanoma patients treated at Roswell Park Memorial Institute from 1972 to 1978 found that 125/700 (18%) developed brain metastases [2]. In contrast, an autopsy study reported melanoma as the most commonly encountered source of brain metastasis. The incidence of melanoma brain metastasis was recorded as 91%, followed by breast (37%) and lung (34%) cancer [3]. An important event during brain metastasis, at least for some cancers, is the extravasation of cancer cells from the vascular lumina across the blood-brain barrier, which consists of the vascular endothelium and surrounding cells, into the brain parenchyma [4]. Not all cancers seem to share the same propensity to cross the blood-brain barrier. Of note, microRNA-containing vesicles capable of destroying the blood-brain barrier have recently been identified as a mechanism in breast cancer [5].

With particular reference to melanoma, the biology and mechanisms of brain metastasis have received little attention in the literature. Recently, *in vitro* and *in vivo* studies by Lugassy and Barnhill demonstrated the capacity of melanoma cells to spread to local and regional (and perhaps even distant) sites via migration along the external (abluminal) surfaces of vascular channels without intravasation [6]. This revolutionary new paradigm of tumour spread has been termed extravascular migratory metastasis and may partially explain why brain metastases are so frequently encountered in melanoma.

In order to better understand the characteristics of this patient population, we retrospectively analysed 193 patients with melanoma brain metastases, diagnosed between 1990 and 2015.

## Patients and Methods

### Study population

Patients were admitted at the University Hospital Zurich and some provided written informed consent in accordance with the Declaration of Helsinki. As not all patients were able to do so, Ethics Commission Zurich, Switzerland, also approved the here reported melanoma study (approval number KEK-ZH-Nr. 2014–0193 and Stv. 16–2007, amendment 2014).

The electronic database of the Department of Pathology at the University Hospital Zurich, existing since 1990, was searched for melanoma patients with histologically confirmed brain metastasis between 1990 and 2015. In the clinical electronic database, which dates to 1995, patients with histologically confirmed melanoma brain metastasis were further investigated for sex, age at diagnosis of primary melanoma, the sequence and extent of metastatic spread.

In addition, we were interested in identifying patients without lymphatic spread, indicating primary hematogenous or extravascular migratory dissemination. The primary melanoma was characterised according to anatomic location, subtype, Breslow tumour thickness and Clark Level. Brain metastases were further classified as either solitary or multiple. Since 2009 every initial melanoma metastasis was tested for the *BRAF* mutation status, followed by the *NRAS* mutation analysis in *BRAF* wild-type tumours

Follow-up data were extracted, including the time interval between primary diagnosis and manifestation of brain metastases and survival time. Survival information was collected in collaboration with the Cancer Registry Zurich and Zug, Switzerland.

### Statistical analysis

Statistical analysis was performed using IBM SPSS Statistics, version 21.0 (Armonk, NY: IBM Corp.). Results of the descriptive statistics were expressed as numbers, percentages and median with range. Data were analysed using the binominal test, chi-square test, Mann-Whitney U test and the Kruskal-Wallis test. Overall survival was analysed using Kaplan-Meier curves. Median survival is reported with 95% confidence intervals (CI). Groups were compared using the log-rank test. All statistical tests were two-sided and an exact p-value < 0.05 was considered to be statistically significant.

## Results

### Patients

**Fig 1:**
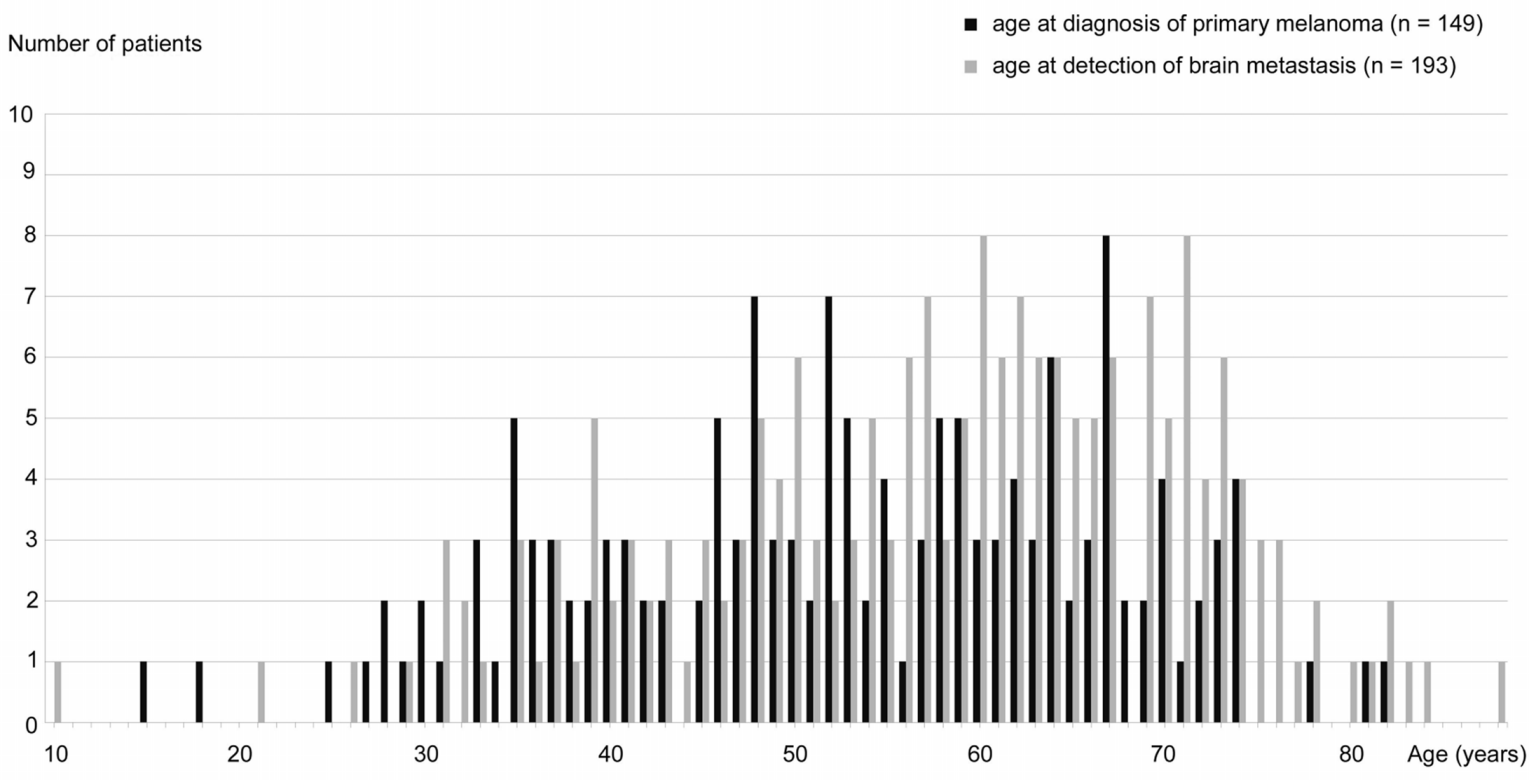
Age distribution. (a) Black: age at diagnosis of primary melanoma (n = 149). (b) Grey: age at detection of brain metastasis (n = 193).

193 patients with melanoma brain metastasis were identified. In this population, there was a significant male predominance (126/193 men (65%) versus 67/193 women (35%); p < 0.001). The median age at diagnosis of primary melanoma was 53.0 years (n = 149; range 15–82 years) (Fig 1A). There was no significant age difference between men and women. The median age at detection of brain metastasis was 60.0 years (n = 193; range 10–88 years) (Fig 1B). There was no significant age difference between men and women.

155 patients could be analysed with respect to the clinical time course. In the majority of patients (n = 108/155; 70%), brain metastasis was detected later than metastasis to other organs. In 34 patients (22%) brain metastases were simultaneous (within 2 months) with metastases to other organs. Eight patients (5%) presented with brain metastasis before metastasis to other organs. 5 patients (3%) harboured only brain metastasis. At the initial diagnosis of brain metastases, 98 of 193 patients (51%) presented with a solitary and 95 of 193 patients (49%) with multiple brain tumours.

15 of 193 patients (8%) had a history of a second primary melanoma.

### Primary melanoma

The back was the most frequent location in males (26 of 90 melanomas; 29%) and the lower extremities in females (14 of 49 melanomas; 29%). Males tended to have melanomas located on the chest and abdomen (p = 0.06), whereas location on the arms was observed more frequently in females (p = 0.07). Mucosal melanomas were significantly more common in women (p = 0.02), taking into account that vaginal primary melanoma accounted for 2 of 5 mucosal locations (40%) in women (Table 1).

**Table 1:** Location of the primary melanoma according to gender.

	Total cases	Male	Female	p-value
	n = 139	n = 90	n = 49	(male vs female)
	(100%)	(100%)	(100%)	
Back	35 (25%)	26 (29%)	9 (18%)	n.s.
Legs	28 (20%)	14 (16%)	14 (29%)	n.s.
Head and neck	26 (19%)	20 (22%)	6 (12%)	n.s.
Chest and abdomen	23 (17%)	19 (21%)	4 (8%)	0.06
Arms	20 (14%)	9 (10%)	11 (22%)	0.07
Mucosa	6 (4%)	1 (1%)	5 (10%)	0.02
Iris	1 (1%)	1 (1%)	0 (0%)	n.s.

n.s., not significant.

Nodular melanoma (NM) was the most common subtype (44 of 105; 42%), followed by superficial spreading melanoma (SSM) in 30 of 105 (29%). There was no significant gender difference in these two subtypes (Table 2).

**Table 2:** Subtypes of primary melanoma according to gender.

	Total cases	Male	Female	p-value
	n = 105	n = 72	n = 33	(male vs female)
	(100%)	(100%)	(100%)	
NM	44 (42%)	33 (46%)	11 (33%)	n.s.
SSM	30 (29%)	22 (31%)	8 (24%)	n.s.
ALM	7 (7%)	3 (4%)	4 (12%)	n.s.
LMM	7 (7%)	4 (6%)	3 (9%)	n.s.
Mucosal	6 (6%)	1 (1%)	5 (15%)	0.01
Other variants	11 (11%)	9 (13%)	2 (6%)	n.s.

NM, nodular melanoma; SSM, superficial spreading melanoma; ALM, acral lentiginous melanoma; LMM, lentigo maligna melanoma; n.s., not significant.

**Fig 2:**
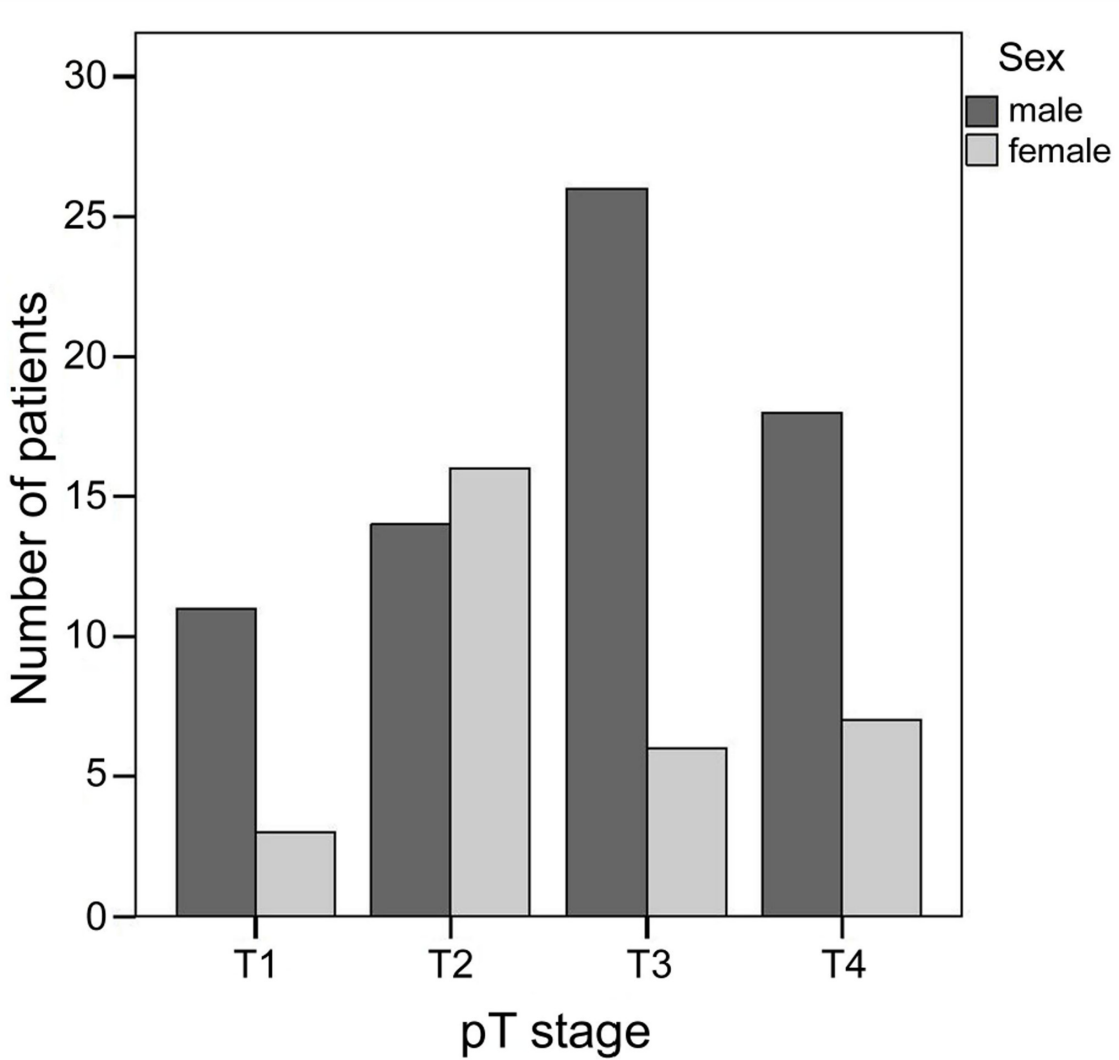
Comparison of T stages between males and females.

In 101 melanomas, the T classification of the AJCC/UICC staging system [7] was reported. 14 melanomas (14%) were classified as T1 (≤ 1.00 mm thickness), 30 (30%) as T2 (1.01–2.00 mm thickness), 32 (32%) as T3 (2.01–4.00 mm thickness) and 25 (25%) as T4 (> 4 mm thickness). There is a significant gender difference with regard to the T stages (p = 0.02). Males account for 11 out of 14 (79%) T1 melanomas, 14 out of 30 (47%) T2 melanomas, 26 out of 32 (81%) T3 melanomas and 18 out of 25 (72%) T4 melanomas (Fig 2).

**Fig 3:**
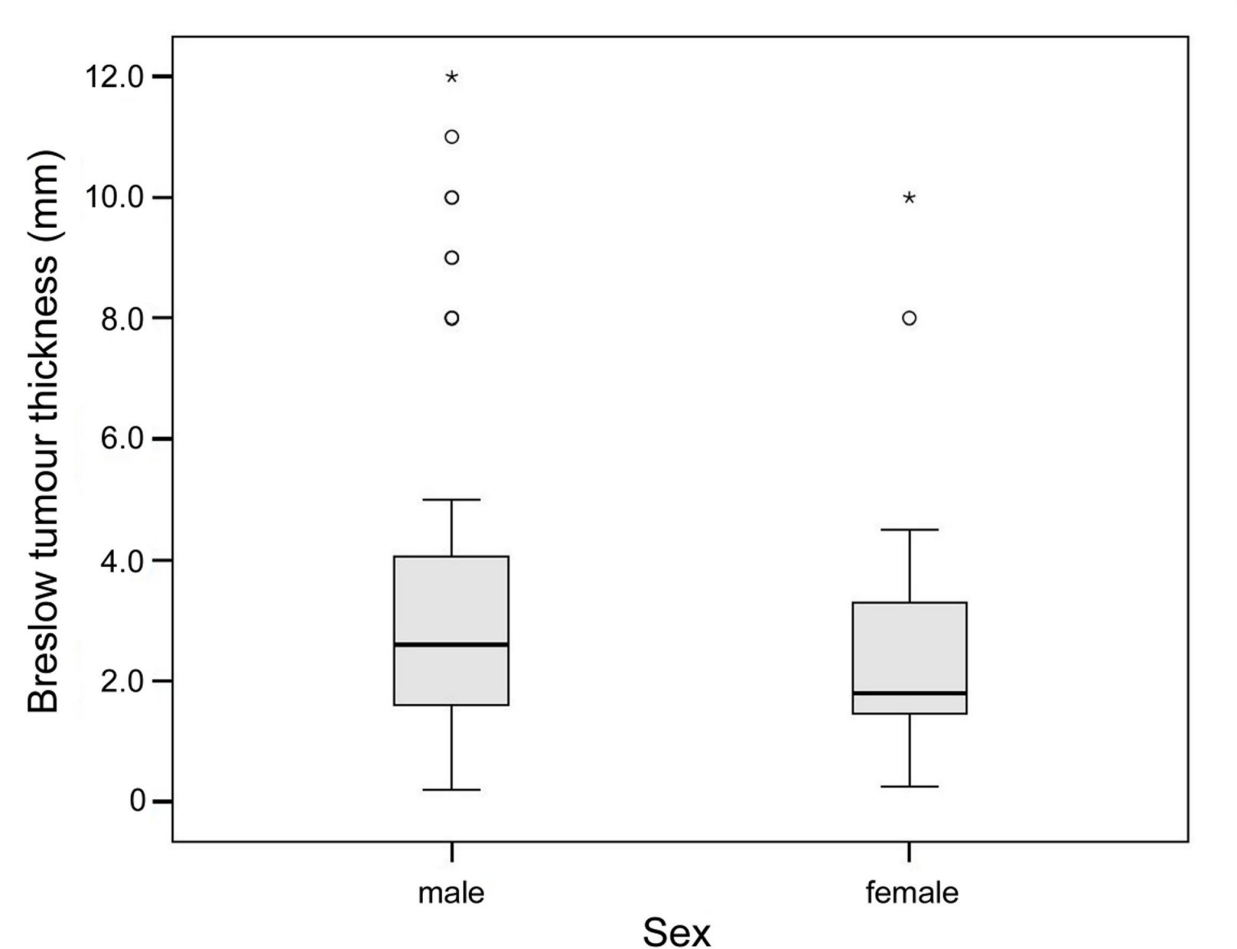
Comparison of Breslow tumour thickness between males and females.

Breslow tumour thickness was reported in 99 primary melanomas and had median of 2.3 mm and a range of 0.2 to 12.0 mm. There was no significant difference in Breslow tumour thickness between men and women (Fig 3). Median tumour thickness was 2.6 mm in men (n = 67; range 0.2–12.0 mm) and 1.8 mm in women (n = 32; range 0.3–10.0 mm). Clark level was reported in 94 melanomas: 3 melanomas (3%) were classified as Clark Level II, 23 (25%) as Clark Level III, 60 (64%) as Clark Level IV and 8 (9%) as Clark Level V.

Thirty-two of 193 (17%) patients had an unknown primary melanoma, 20 (63%) thereof were males.

### Mutation status

The mutation status was known in 55 of 193 (28%) patients. In 22 (40%) patients melanomas were *BRAF* wild-type, 23 (42%) showed a *BRAF* mutation, and 10 (18%) a *NRAS* mutation. There was a significant difference in the age at detection of brain metastasis according to the mutational status (p = 0.01). The median age at detection of brain metastasis was 56.0 years in *BRAF* mutated tumours (range 35–71 years), 59.5 years in *NRAS* mutated tumours (range 35–76 years) and 69.5 years in *BRAF* wild-type tumours (range 40–75 years).

### Comparing patients with and without lymph node metastasis

Information about lymph node involvement was available in 126 patients. In this cohort, 108 (86%) patients showed lymph node metastasis at the time when brain metastasis was diagnosed, whereas 18 (14%) did not. Among 58 patients with positive nodes, 24 patients (41%) were classified as N1, while the N2 and N3 stages contained 17 patients each (29%). Metastases, apart from lymph nodes and brain, involved the skin, lung, liver, kidney, bone, and other sites in 124 of 193 patients (64%) in the course of their disease.

We found a significant difference in melanoma subtype between patients with and without lymph node metastasis (p = 0.006). All 26 patients with SSM manifested lymphatic spread concurrent with the diagnosis of brain metastasis. In contrast, the absence of lymph node metastasis was found in 3 out of 4 lentigo maligna melanomas (LMM) and in 10 out of 42 NM (24%). NM accounted for the largest group of lymph node negative patients (10/16; 63%) (Table 3). There was no significant difference between patients with and without lymph node metastasis with respect to Breslow tumour thickness. However, there was a significant difference in Breslow tumour thickness between malignant melanoma of NM and SSM types (p < 0.001). Breslow tumour thickness ranged from 1.4 to 9.0 mm in NM (n = 39; median 3.6 mm) and from 0.2 to 4.9 mm in SSM (n = 27; median 1.5 mm).

**Table 3:** Subtypes of primary melanoma according to the presence or absence of lymph node metastasis.

	Total cases	N+	N-	p-value
	n = 90	n = 74	n = 16	(N+ vs N-)
NM	42 (100%)	32 (76%)	10 (24%)	n.s.
SSM	26 (100%)	26 (100%)	0 (0%)	0.004
ALM	6 (100%)	6 (100%)	0 (0%)	n.s.
LMM	4 (100%)	1 (25%)	3 (75%)	0.02
Mucosal	3 (100%)	2 (67%)	1 (33%)	n.s.
Other variants	9 (100%)	7 (78%)	2 (22%)	n.s.

NM, nodular melanoma; SSM, superficial spreading melanoma; ALM, acral lentiginous melanoma; LMM, lentigo maligna melanoma; N+, lymph node positive melanoma; N-, lymph node negative melanoma; n.s., not significant.

History of a second melanoma was significantly associated with the absence of lymph node metastasis (p = 0.003).

We found no significant difference between patients with and without lymph node metastasis with respect to sex, age at detection of primary melanoma or brain metastasis, occurrence of metastases apart from the lymph nodes and the brain, number of brain metastases, Clark Level and mutation status.

We found no significant difference in the location of primary melanoma with respect to age at detection of brain metastasis.

### Comparing cases with known and unknown primary melanoma

There was no significant difference between patients with known and unknown primary melanoma regarding sex, age at detection of brain metastasis, presence or absence of lymph node metastasis, occurrence of metastases apart from the lymph nodes and the brain, number of brain metastases and mutation status.

Compared to other primary melanomas, lymph node and visceral (other than brain) metastases associated with unknown primary melanomas showed a significantly different clinical time course (p = 0.04). In unknown primary melanomas, they were more commonly synchronous (within 2 months) with brain metastases (9/19 cases; 47%). In contrast, most known primary melanomas (99/136; 73%) manifested the sequential development of lymph node or visceral metastases, later followed by brain metastases as the final step in tumour progression (for details consult S1 Dataset).

### Follow-up data

Survival and follow-up data were considered as reliable only if patients were dead or reported alive within one year before the end of data collection. 166/193 (86%) patients fulfilled these criteria and were included in the statistical survival analysis. 17 of these 166 well documented patients (10%) were still alive in 2015 or 2016. Of those, 2 patients had a melanoma of unknown primary. In the group of patients with unreliable follow-up data, 21/27 (78%) carried the diagnosis of brain metastases 10 years or longer ago. Considering the short median survival after diagnosis of brain metastasis (0.5 years) in patients with reliable follow-up, it is probable that most patients with poor follow-up data are also dead (for details consult S2 Dataset).

The median age at death was 61.2 years (n = 149; range 24–89 years). There was no significant age difference between men and women. The median time between diagnosis of primary melanoma and of brain metastasis was 3.4 years (n = 149; range 0–33.2 years) without a significant difference between men and women. Breslow tumour thickness neither significantly influenced this time interval. The median survival after detection of brain metastasis was 0.47 years (95% CI 0.39 to 0.66 years) with a borderline significantly better prognosis for women (n = 56; median survival 0.71 years; 95% CI 0.43 to 1.0 years) compared to men (n = 110; median survival 0.43 years; 95% CI 0.32 to 0.56 years; p = 0.05). Breslow tumour thickness did not influence survival after brain metastasis. For the 17 patients who are still alive, the median follow-up after diagnosis of brain metastasis was 1.9 years, range 0.2–13.5 years.

## Discussion

In this study of 193 melanoma patients with brain metastases, men (65%) were more frequently affected than women (35%). Gender is widely accepted as an independent prognostic factor in predicting outcome for patients with cutaneous melanoma [8–10]. The predominance of men with brain metastases is congruent with a previous study showing increased risk in men for visceral metastases and a correspondingly higher mortality rate [10]. As men have additional unfavourable primary melanoma characteristics such as increased tumour thickness, ulceration, axial location and older age [11], there is uncertainty as to whether this gender difference is due to behavioural factors or an inherent biological difference. Negligence with respect to health care is often suggested to explain this gender difference for thick melanomas, as we observed in this study. Aside from nodal status, Breslow tumour thickness remains among the most important prognostic indicators for melanoma. Additionally, tumour thickness has been reported as the only prognostic confounder affecting gender survival differences in one study [10]. Therefore, our finding of a significant gender difference with regard to T stages (p = 0.02) is of considerable interest. Of special note is the high percentage (14 of 101; 14%) of T1 stage melanomas with clear-cut male predominance (79% men). This observation is supported by previous studies indicating that female survival advantage relates at least partially to biological differences, either tumour or host related [10, 12–14]. Interestingly a recent study demonstrated that sex differences in melanoma survival is not related to mitotic rate of the primary tumour [15]. In our study men displayed a borderline significantly shorter median survival after detection of brain metastasis compared to women (p = 0.05). This observation underscores a survival benefit in women. However, interpretation of survival data is limited by the different treatment regimens in this study cohort. Vice versa, the prognostic value of the current dataset is enhanced by the fact that all patients underwent surgery at least once for histologic confirmation of brain metastasis.

Thin T1 melanomas have an excellent prognosis; the range of melanoma associated deaths reported in the literature however is substantial (1–8% at 10 years [7, 16–18]). In addition, many studies regarding melanoma recurrence and survival are reported with end points of 5 to 10 years. Especially for thin melanomas, this period of surveillance is too short since approximately 10% of lesions show recurrence beyond 10 years [19]. Many large multicentre survival studies have failed to make adjustments to differentiate between death from all causes and death due to disease. In such cases, longer observation periods may result in falsely high results. Accordingly, our results with the endpoint of brain metastases provide more robust survival information. Importantly, our data illustrate that there is a significant male predominance for thin melanomas with subsequent brain metastasis.

Interestingly, we found 17% of patients with unknown primary melanomas. This incidence is much higher than previously reported (3.2% [20] and 0.9% [21], respectively). In routine clinical practice, metastatic melanoma with unknown primary is considered to originate from completely regressed primary melanomas. From our experience, especially in immune compromised patients after organ transplantation [22], we fully recognise the importance of immune surveillance in preventing the development and progression of cancer [23]. Regression is common in melanoma, particularly in thin lesions with radial growth phase. Its incidence across all melanoma tumour thicknesses amounts to 10–35% [24], and up to 58% in thin melanomas (< 0.75 mm) [25]. Regression has the following histologically defined phases: An early phase characterised by an area of tumour accompanied by tumour infiltrating lymphocytes (TILS) and associated with necrosis of tumour cells. In the intermediate phase, tumour gradually replaced by granulation tissue and partly pigmented macrophages. In the late phase, the histological picture is dominated by scar with partly pigmented macrophages. In the final phase, melanoma cells are completely replaced by scar tissue, thus precluding the histological diagnosis of melanoma [26]. Clark et al. have shown in 1989 that TILS are associated with good prognosis and many authors have confirmed this observation since then [9, 24, 25]. On the contrary, prognostic significance of regression, particularly in patients with thin melanomas, has been lately controversially debated. Some studies have found that the presence of regression is a poor prognostic indicator [26–31], while others found that it has no effect or even a benefit on the risk of recurrence or survival [32–35]. Possible limitation of some multi-institutional and multinational studies might be methodical inconsistencies, for example not performing slide reviews. Additionally, assessment of regression has high interobserver variability [25, 28, 36]. Interestingly several studies have shown that thin melanomas with extensive late regression have higher risk for metastatic disease [26, 29–31]. Our results coincide with these findings.

In our patients with unknown primaries, which at least partially represent completely regressed melanomas, there was a significant difference in the pattern of metastatic spread (p = 0.04). Compared to other primary melanomas, lymph node and visceral (other than brain) metastases associated with unknown primary melanomas were more commonly synchronous (within 2 months) with brain metastases (in 9/19 cases; 47%), indicating an aggressive clinical course. In contrast, most known primary melanomas (99/136; 73%) manifested the sequential development of lymph node or visceral metastases, later followed by brain metastases as the final step in tumour progression. However, this difference might result from a diagnostic bias because all patients included in the study had brain metastasis. Unfortunately, a prospective approach to confirm our results is not feasible.

Absence of lymph node metastases was found in 16 out of 90 (18%) melanoma patients at the time of initial diagnosis of brain metastasis. Interestingly, most of those patients had primary melanomas of nodular type (10/16; 63%) with Breslow tumour thickness between 1.7 and 5.0 mm. In contrast, all 26 patients with SSM type melanoma showed lymph node metastases, and their tumours were significantly thinner when compared to NM type melanomas (p < 0.001) (Table 3). These observations suggest that tumour thickness plays an important role in primary hematogenous metastasis.

Furthermore, patients without lymphatic spread had significantly greater numbers of multiple primary melanomas (p = 0.003), indicating a special genetic and/ or immunological status in this subgroup.

Interestingly, a recent study identified more frequent *BRAF* or *NRAS* mutations in brain metastases compared to primaries and metastasis at other sites [37]. The *NRAS* mutation was reported as an independent prognostic factor in metastatic melanoma [38]. Furthermore, melanomas harbouring *NRAS* or *BRAF* mutations were associated with a trend towards worse survival and a greater likelihood of brain metastases at the time of initial diagnosis [37]. In our cohort the *BRAF* and *NRAS* mutation status was documented in 55 of 193 patients (29%): 22 patients (40%) manifested *BRAF* wild-type, 23 (42%) *BRAF* mutations, and 10 (18%) *NRAS* mutations. The latter results are congruent with those found in the literature [39]. Depending upon the mutational status of the patient, there was a significant difference in patient age at the time of detection of brain metastases (p = 0.01). The median age at detection of brain metastasis was 56.0 years in *BRAF* mutated tumours, 59.5 years in *NRAS* mutated tumours and 69.5 years in *BRAF* wild-type tumours. This age difference between patients with *BRAF* and *NRAS* mutations is consistent with that reported in the literature [38, 40–41].

## Conclusions

What emerges from this study is a clearer picture of factors associated with brain metastasis in patients suffering from malignant melanoma. Accordingly, our results provide a framework for the reliable stratification of metastatic potential, which in turn should facilitate rational clinical planning and treatment.

Of special interest are the higher incidence of thin melanomas (14%) with male predominance (79%) and the higher incidence of unknown primaries (17%) (representing at least partly completely regressed melanomas) with metastatic disease if compared with the literature. These findings illustrate the importance of gender (genetic or epigenetic) and immune surveillance in melanoma progression.

## Supporting Information

S1 Dataset Underlying participant-level data of the study. (XLSX)

S2 Dataset Follow-up data of the study. (XLSX)
